# MicroRNA-137 is downregulated in glioblastoma and inhibits the stemness of glioma stem cells by targeting RTVP-1

**DOI:** 10.18632/oncotarget.928

**Published:** 2013-04-09

**Authors:** Ariel Bier, Nis Giladi, Noam Kronfeld, Hae Kyung Lee, Simona Cazacu, Susan Finniss, Cunli Xiang, Laila Poisson, Ana C. deCarvalho, Shimon Slavin, Elad Jacoby, Michal Yalon, Amos Toren, Tom Mikkelsen, Chaya Brodie

**Affiliations:** ^1^ Everard and Mina Goodman Faculty of Life Sciences, Bar-Ilan University, Ramat-Gan, Israel; ^2^ Davidson Laboratory of Cell Signaling and Tumorigenesis, Hermelin Brain Tumor Center, Department of Neurosurgery, Henry Ford Hospital, Detroit MI USA; ^3^ Department of Public Health Sciences, Tel Aviv, Israel; ^4^ The International Center for Cell Therapy and Cancer Immunotherapy (CTCI), Tel Aviv, Israel; ^5^ Pediatric Hemato-Oncology, The Edmond and Lilly Safra Children's Hospital, Sheba Medical Center, Tel-Hashomer and The Sackler School of Medicine, Tel-Aviv University, Israel

**Keywords:** Glioma stem cells, self renewal, miR-137, RTVP-1, CXCR4

## Abstract

Glioblastomas (GBM), the most common and aggressive malignant astrocytic tumors, contain a small subpopulation of cancer stem cells (GSCs) that are implicated in therapeutic resistance and tumor recurrence. Here, we study the expression and function of miR-137, a putative suppressor miRNA, in GBM and GSCs. We found that the expression of miR-137 was significantly lower in GBM and GSCs compared to normal brains and neural stem cells (NSCs) and that the miR-137 promoter was hypermethylated in the GBM specimens. The expression of miR-137 was increased in differentiated NSCs and GSCs and overexpression of miR-137 promoted the neural differentiation of both cell types. Moreover, pre-miR-137 significantly decreased the self-renewal of GSCs and the stem cell markers Oct4, Nanog, Sox2 and Shh. We identified RTVP-1 as a novel target of miR-137 in GSCs; transfection of the cells with miR-137 decreased the expression of RTVP-1 and the luciferase activity of RTVP-1 3'-UTR reporter plasmid. Furthermore, overexpression of RTVP-1 plasmid lacking its 3'-UTR abrogated the inhibitory effect of miR-137 on the self-renewal of GSCs. Silencing of RTVP-1 decreased the self-renewal of GSCs and the expression of CXCR4 and overexpression of CXCR4 abrogated the inhibitory effect of RTVP-1 silencing on GSC self-renewal. These results demonstrate that miR-137 is downregulated in GBM probably due to promoter hypermethylation. miR-137 inhibits GSC self-renewal and promotes their differentiation by targeting RTVP-1 which downregulates CXCR4. Thus, miR-137 and RTVP-1 are attractive therapeutic targets for the eradication of GSCs and for the treatment of GBM.

## INTRODUCTION

Glioblastoma (GBM), the most common and malignant primary brain tumors, are highly invasive, proliferative and vascularized [1]. The median survival of patients with GBM, following tumor resection and treatment with radiation and chemotherapy, is less then 16 months [1-3]. The dismal prognosis of these patients is mainly due to the infiltrative nature of GBM and the resistance of residual tumor cells to current therapeutic modalities [4, 5].

GBM contain a small subpopulation of self-renewing and tumorigenic cancer stem cells (glioma stem cells, GSCs) that are implicated in tumor infiltration, resistance to conventional therapies and tumor recurrence [4,5]. Therefore, understanding the mechanisms associated with the stemness and oncogenic features of these cells is essential for the development of therapeutic approaches that can eradicate GSCs and may provide the basis for the development of novel therapeutic approaches to treat GBM patients [6].

MicroRNAs (miRNAs) are small non-coding RNAs that regulate gene expression by either degradation or repression of mRNA translation [7, 8]. miRNAs cause gene silencing by partial sequence homology and thus a single miRNA can have hundreds of targets and therefore regulate diverse cellular functions [8]. Indeed, alteration in miRNA expression has been associated with various disorders including cancer [9, 10].

Recently, various studies have highlighted the important of miRNA deregulation in several aspects of the pathogenesis of GBM including cell cycle control, invasion, migration, resistance to chemotherapy and radiotherapy and cell apoptosis (11-21). Specific miRNAs control some of the core signaling pathways in GBM such as EGFR signaling and those related to p53, PTEN/PI3K/AKT and the notch pathways. Some miRNAs such as miR-124, miR-34a and miR-145 have been identified as tumor suppressor miRNAs [11-13], whereas others such as miR-10b have been identified as oncomiRs [14].

miRNAs also play important roles in the self-renewal and pluripotency of stem cells [15] and in the functions of GSCs [16]. Indeed, miR-124, miR-145, miR-128, miR-9, miR-9*, miR-17, miR-302-367 cluster and miR-34a have been shown to induce differentiation of GSCs and to impair their stemness characteristics. MiR-125b and miR-9* have been implicated in the chemo/radio-resistance of GSCs and miR-124, miR 302-367 cluster and miR-138 inhibit the apoptosis pathway in these cells [11, 17-21].

Hypermethylation of CpG islands located in the promoter regions of tumor suppressor genes is now firmly established as an important mechanism of gene inactivation. CpG island hypermethylation has been described in almost every tumor type and many cellular pathways are inactivated by this type of epigenetic pathway [22]. Recent studies have shown that the downregulation of various miRNAs in some tumors is also mediated by CpG island hypermethylation [23].

miR-137 is a brain-enriched miRNA which has been shown recently to be downregulated in GBM and is considered a putative tumor suppressor miRNA (24). In glioma cell lines, miR-137 induces cell cycle arrest and suppresses migration and invasion. Moreover, miR-137 induces neuronal differentiation of mouse NSCs and in primary GBM culture [24, 25].

Here, we examined the expression of miR-137 in glial tumors and GSCs and its role in self-renewal and differentiation of these cells. In addition, we identified RTVP-1 as a novel target of miR-137 and demonstrated its effects on the self-renewal of GSCs downstream of miR-137 and upstream of CXCR4.

## RESULTS

### Expression of miR-137 in GBM specimens and its methylation status

**Figure 1:**
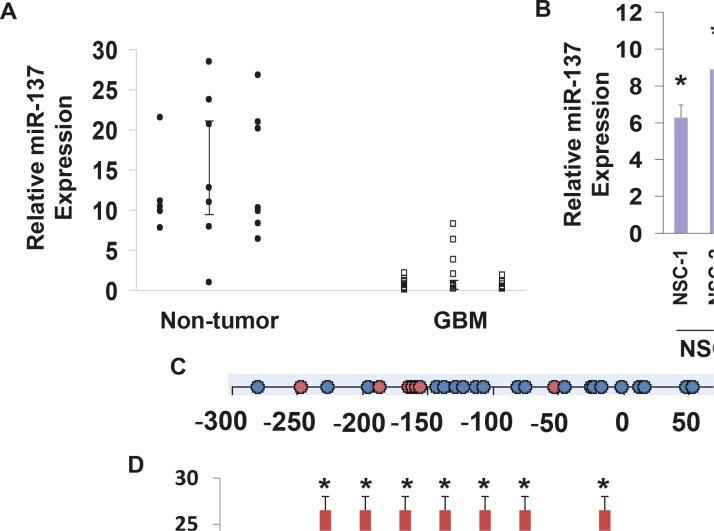
Expression of miR-137 in glial tumors and GSCs The expression of miR-137 was determined in glial tumors and normal brains using real-time PCR (A). Data from individual human tissues are presented with the median and interquartile range noted. Age adjusted t-test, P=0.001. Results are normalized relative to the levels of S12 mRNA and are presented relative to a reference sample (p<0.005 for all tumor as compared to non-tumor specimens). Expression of miR-137 was also determined for ten GSCs compared to two NSCs (B) *P < 0.001. Methylation of miR-137 was determined in twenty five GBM and seven normal brain specimens. An amplicon, starting 300 bp upstream of and ending 167 bp downstream of the pre-miR-137 start codon was used for the methylation analysis, covering 32 CpG sites. Circles represent CpG site (C). Methylation was determined by Sequenom mass spectrometry for each CpG position in the specific amplicon (D). *P < 0.01.

We first examined the expression of miR-137 in GBM compared to normal brain specimens and in GSCs compared to NSCs using real-time PCR. As presented in Figure 1A, the expression of miR-137 was significantly lower in the GBM specimens that were examined compared to normal brains. In addition, the expression of miR-137 was significantly lower in all the GSCs compared to human NSCs (Figure 1B).

miR-137 contains CpG islands in its promoter, therefore, hypermethylation can play a role in the downregulation of this miRNA expression in the tumor specimens. We analyzed a region of CpG islands in miR-137 promoter (Figure 1C) and found that this region is hypermethylated in GBM compared to normal brain specimens (Figure 1D). Therefore, changes in methylation may contribute to the downregulation of miR-137 in GBM.

### Role of miR-137 in the differentiation and self-renewal of NSCs and GSCs

**Figure 2:**
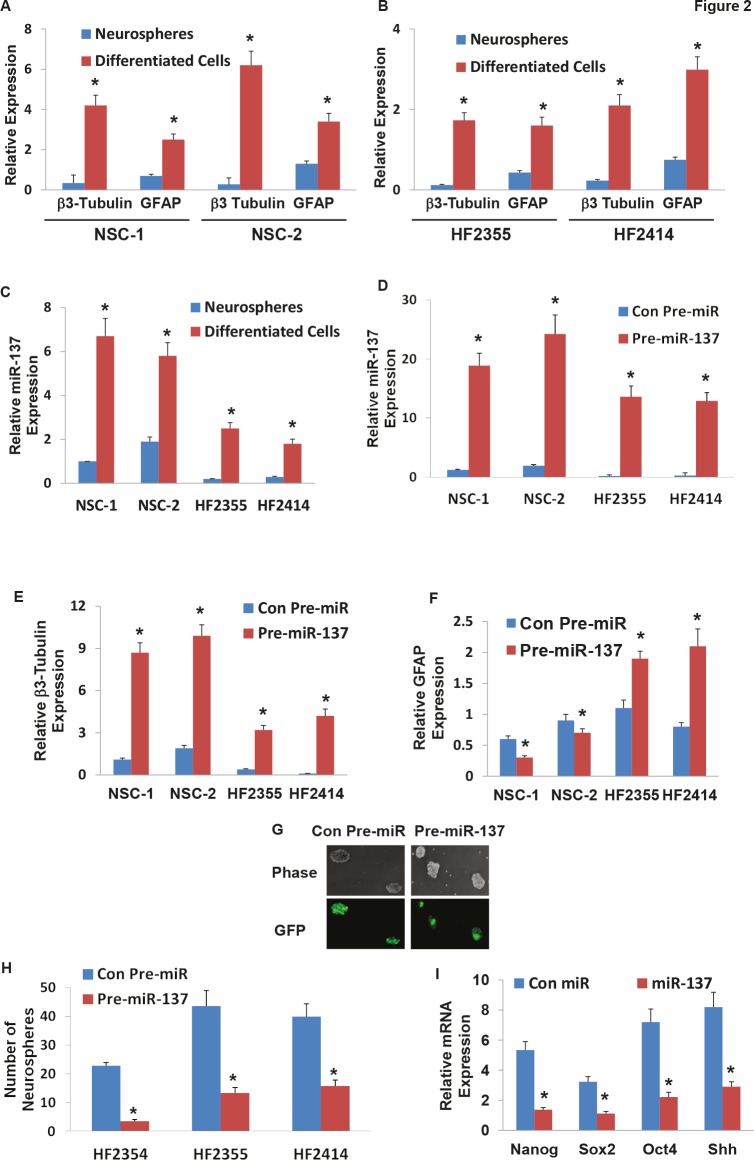
Roles of miR-137 in NSC and GSC differentiation, GSC self-renewal and stemness markers Two NSCs (A) and GSCs (HF2355 and HF2414) (B) were maintained as neurospheres or were induced to differentiate by plating them on laminin in NM medium. The expression of β3-tubulin (A,B), GFAP (A,B) and miR-137 (C) was determined after 10 days of treatment using real-time PCR. NSCs (NSC-1 and NSC-2) and GSCs (HF2355 and HF2414) were transduced with a lentivirus vector expressing pre-miR-137-GFP or control pre-miR-GFP. After 1 week the transduction efficacy in the neurospheres was determined using real-time PCR analysis (D) and fluorescence microscopy (G). The differentiation of the NSCs (E,F) and the GSCs (F,F) was determined by analyzing the expression of β3-tubulin (E) and GFAP (F) using real-time PCR. Self-renewal assay was performed 14 days post transduction as described in the methods (H). The expression of the stemness markers, Nanog, Sox2, Oct4 and Shh was determined in GSCs expressing pre-miR-137 or control pre-miR using real-time PCR (I). The results are representative of three different experiments that gave similar results (G) or are the mean ± SE of three different experiments.*P<0.05.

Recent studies indicated that miR-137 plays a role in the neuronal differentiation of mouse NSCs and glioma cell lines (24). We further examined the role of miR-137 in the differentiation of human NSCs and the differentiation and self-renewal of GSCs. We first examined the expression of miR-137 in NSCs and GSCs that were grown as neurospheres or were induced to differentiate. NSCs were plated on laminin-coated plates and were differentiated by incubation with NM media. As presented in Figure 2A, differentiation of the human NSCs resulted in increased levels of the neuronal marker β3 tubulin and the astrocytic marker GFAP. The differentiated cells also exhibited a large increase in the expression of miR-137 as compared to control undifferentiated neurosphere cultures (Figure 2C). The differentiation of the GSCs was performed under similar conditions and resulted in increased expression of the differentiation markers, GFAP and β3-tubulin (Figure 2B). Similarly, the differentiation of these cells was also followed by upregulation of miR-137 albeit to a lower level than its expression in the differentiated NSCs (Figure 2C).

We then examined the effects of miR-137 on the differentiation of NSCs using lentivirus vector expressing pre-miR-137. As presented in Figure 2D, the transduction of the NSCs resulted in high levels of miR-137 as determined by real-time PCR and in a significant increase in the neuronal differentiation of these cells (Figure 2E) with some decrease in their astrocytic differentiation (Figure 2F). We then examined the effect of miR-137 on the differentiation of GSCs using two GSCs, HF2355 and HF2414 that express low levels of this miRNA. The GSCs were transduced with a lentivirus vector expressing pre-miR-137-GFP or control pre-miR-GFP vectors. Transduction of these cells resulted in an increased level of miR-137 as determined by real-time PCR (Figure 2D) and by increased GFP expression in the transduced HF2414 neurospheres as analyzed by fluorescence microscopy (Figure 2G). Transduction of the GSCs with pre-miR-137 induced an increase in the neuronal differentiation of these cells (Figure 2E) and some increase in the expression of GFAP (Figure 2F).

The ability to form neurospheres is one of the hallmarks of GSC stemness and correlates with the tumor-initiating and tumorigenic potential of these cells. We therefore evaluated the effect of miR-137 on the neurosphere formation of the GSCs. We employed three different GSCs, HF2354, HF2355 and HF2414 that express low levels of this miRNA and transduced them with a lentivirus vectors expressing pre-miR-137 or control pre-miR. Transduction of the GSC neurospheres with lentivirus vector expressing pre-miR-137 significantly decreased the secondary neurosphere formation by 6.6-fold, 3.3-fold, and 2.8-fold, respectively, compared with GSCs that were transduced with a lentivirus vector expressing a control pre-miR (Figure 2H).

Additionally, we showed that miR-137 decreased the expression of the stemness markers, Nanog, Oct4, Sox2 and Shh in the transduced HF2355 GSCs (Figure 2I) and in the HF2414 and HF2354 GSCs (data not shown).

Transduction of the GSCs with pre-miR-137 did not induce cell death (data not shown) and therefore the decreased neurosphere formation was not due to this process.

### RTVP-1 is a novel target of miR-137

To delineate the mechanism by which miR-137 inhibited the self-renewal of the GSCs, we searched for miR-137 target genes using “miRwalk”(www.ma.uni-heidelberg.de/apps/zmf/mirwalk/), a database of predicted and published microRNAs, and found several putative miR-137 target genes that may play a role in GSC self-renewal. We focused on RTVP-1 since we recently demonstrated that the expression of this gene is correlated with the degree of malignancy of astrocytic tumors and that it is involved in the regulation of the growth, survival and invasion of glioma cells [36, 37].

**Figure 3:**
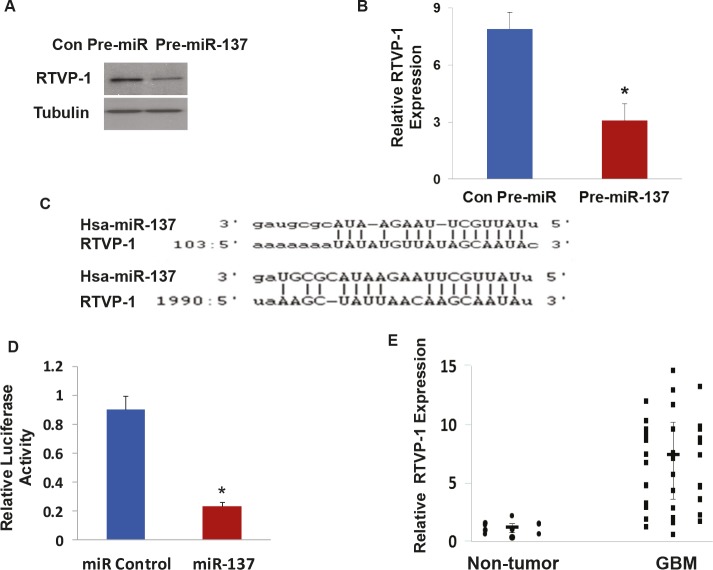
RTVP-1 is a novel target of miR-137 The HF2354 GSCs were transduced with a lentivirus vector expressing pre-miR-137 or a Con-pre-miR. The expression of RTVP-1 was determined using Western blot analysis (A) and real-time PCR (B). Sequence alignment of RTVP-1 3'-UTR and miR-137. Target sites of miR-137 in the RTVP-1 3'-UTR were analyzed using miRwalk and Targetscan (C). U87 glioma cells were transfected with a RTVP-1 3'-UTR -luciferase plasmid followed by transfection with a miR-137 mimic or a control miR. The luciferase activity of the cells was determined after 48 h (D). The expression of RTVP-1 was determined in GBM and normal brain specimens using real-time PCR. Data from individual human tissues are presented with the median and interquartile range noted. Age adjusted t-test, P < 0.001 (E). The results are representative of three different experiments that gave similar results (A) or are the mean ± SE of three different experiments (B,D).*P<0.001.

To confirm that RTVP-1 is a miR-137 target gene, we transduced the HF2354 GSCs with a lentivirus vector expressing pre-miR-137 and examined the effect of this treatment on the expression of RTVP-1. As presented in Figure 3A and 3B, transduction of the HF2354 cells with pre-miR-137 significantly decreased the expression of both RTVP-1 protein and mRNA, respectively compared to cells transduced with a control pre-miR.

We then examined if RTVP-1 is a direct target of miR-137. As presented in Figure 3C, miR-137 appears to have a strong target bias for the 3'-untranslated region (3'-UTR) of RTVP-1; having two predictive seed sequences in this region (Figure 3C). Using a luciferase reporter plasmid in which the 3'-UTR of the RTVP-1 was cloned downstream of luciferase gene (pEZX-MT01:: *RTVP-1*-3'UTR), we demonstrated that miR-137 mimic significantly decreased the luciferase activity of this construct in U87 glioma cells by 4-fold as compared with a control miRNA (Figure 3D).

In addition, real-time PCR analysis of the same GBM and normal brain specimens, that were analyzed for the expression of miR-137 (Figure 1A), demonstrated an inverse pattern of RTVP-1 expression; upregulation in GBM compared to normal brains (Figure 3E). Collectively, these findings strongly indicate that RTVP-1 is a direct target gene of miR-137 in GBM.

### RTVP-1 mediates the inhibitory effect of miR-137 on the self-renewal of GSCs

**Figure 4:**
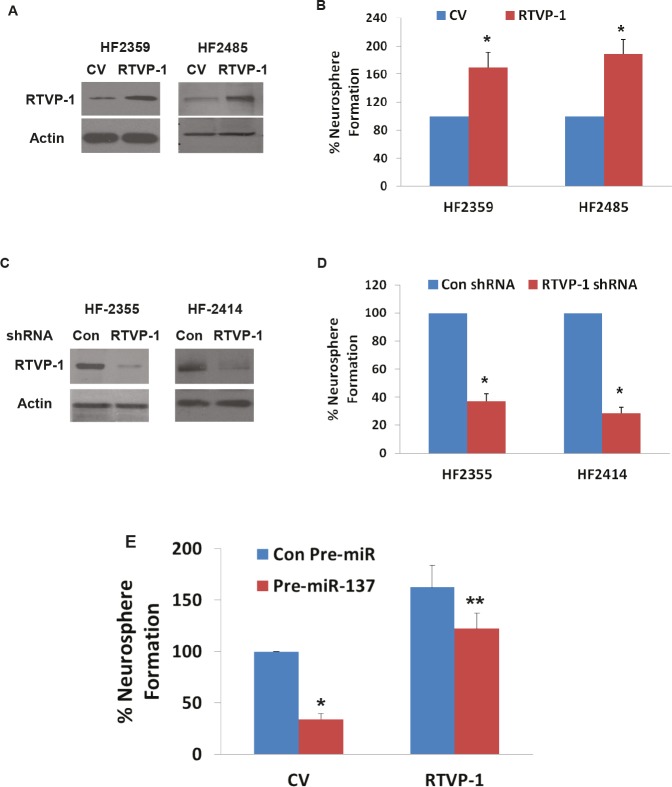
RTVP-1 regulates the self-renewal of GSCs and mediates miR-137 effect GSCs (HF2359 and HF2485) were transduced with lentivirus vectors expressing RTVP-1 or a control vector (CV). The expression of RTVP-1 was determined using Western blot analysis (A) and self- renewal assay was performed 14 days post transduction (B). The GSCs HF2355 and HF2414 were transduced with lentivirus vectors expressing RTVP-1 or control shRNAs and the expression of RTVP-1 was determined using Western blot analysis (C). Self-renewal assay was performed 14 days thereafter (D). The HF2355 GSCs were transduced with lentivirus vectors ex pressing RTVP-1 lacking the 3'-UTR or with a control vector (CV) and lentivirus vector expressing pre-miR-137 or a con-pre-miR and self-renewal assay was performed 14 days post transduction (E). The results are representative of four different experiments that gave similar results (A,C) or are the mean ± SE of four different experiments (B,D,E) *P < 0.001 **P < 0.05

We then examined the role of RTVP-1 in the self-renewal ability of GSCs. We used two GSCs, HF2359 and HF2485, which express low level of endogenous RTVP-1 and transduced them with a lentivirus vector expressing RTVP-1 that increased the expression of the RTVP-1 protein in these cells (Figure 4A). The transduction of both HF2359 and HF2485 GSCs with RTVP-1 significantly increased the self-renewal of these cells by 1.6-fold and 2-fold, respectively, compared to GSCs that were transduced with a control lentivirus vector (Figure 4B). We then examined the effect of RTVP-1 silencing on the self-renewal of the HF2355 and HF2414 GSCs that express high levels of this protein. Transduction of the GSCs with lentivirus vectors expressing shRNAs targeting RTVP-1 decreased the expression of this protein (Figure 4C) and inhibited the self-renewal of the transduced GSCs by 2.9-fold and 3.7-fold, respectively, compared with GSCs that were transduced with a lentivirus vector expressing a control shRNA (Figure 4D).

After demonstrating that RTVP-1 promotes the self-renewal ability of GSCs we examined its role in miR-137 effect. In these experiments, the HF2355 GSCs were transduced with a lentivirus vector expressing a RTVP-1 plasmid lacking the 3'-UTR together with a lentivirus vector expressing pre-miR-137 or with a control pre-miR vector. GSCs that were transduced with pre-miR-137 and control vector exhibited a 3-fold decrease in the number of neurospheres formed compared to the GSCs that were transduced with pre-miR-control. In contrast, transduction with lentivirus vector expressing RTVP-1 lacking the 3'-UTR, abrogated the inhibitory effect of miR-137 on the neurosphere formation of the GSCs (Figure 4E).

### CXCR4 is down-regulated in RTVP-1 silenced GSCs and mediates RTVP-1 effects

**Figure 5:**
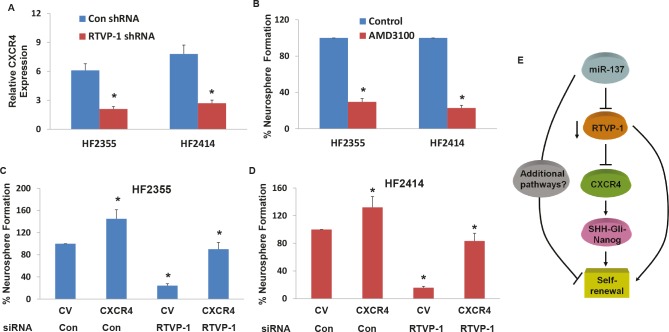
CXCR4 is downregulated in RTVP-1 silenced GSCs and mediates RTVP-1 effects The GSCs HF2355 and HF2414 were transduced with lentivirus vectors expressing control or RTVP-1 shRNAs and the expression of CXCR4 was determined using real-time PCR (A). GSCs (HF2355 and HF2414) were pre-incubated with the CXCR4 inhibitor AMD3100 (50 nM) for 2h prior to the beginning of the assay and every three days thereafter and self-renewal was analyzed after 14 days of treatment (B). The GSCs HF2355 and HF2414 were transduced with lentivirus vectors expressing RTVP-1 shRNA or control shRNA and lentivirus vectors expressing CXCR4 or a control vector (CV). Self-renewal assay was performed 14 days post transduction (C and D). A diagram of the effects of miR-137 on the self-renewal of GSCs by targeting RTVP-1 and the downstream CXCR4 pathway is presented (E). The results are the mean ± SE of four different experiments. *P<0.001.

CXCR4 was recently reported to play a role in the self-renewal of GSCs by disrupting the Shh-GLI-Nanog network [17]. We therefore examined whether this pathway is associated with the effect of RTVP-1 on the self-renewal of GSCs. We found that silencing of RTVP-1 decreased the expression of CXCR4 in the HF2355 and HF2414 GSCs (Figure 5A). To further examine the role of CXCR4 inhibition on GSC self-renewal, we employed the chemical CXCR4 antagonist, AMD3100. The GSCs were pre-treated with AMD3100 (50 nM) for 2 hr prior to the beginning of the assay and it was then added to the cultures every three days thereafter. As demonstrated in Figure 5B, AMD3100 significantly decreased the self-renewal of the GSCs, as was recently reported [17]. We then examined the role of CXCR4 in RTVP-1 effects and found that overexpression of CXCR4 in RTVP-1 silenced HF2355 and HF2414 GSCs abrogated the inhibitory effect of this silencing on the self-renewal of the GSCs (Figures 5C and 5D). Thus, the decreased expression of CXCR4 in the RTVP-1 silenced GSCs partially mediated the decreased self-renewal of these cells.

Collectively, these results demonstrate the importance of miR-137 in controlling the stemness of GSCs by targeting RTVP-1 which in-turn decreases the expression of CXCR4 and its downstream targets Shh and Nanog (Figure 5E).

## DISCUSSION

GBM are the main primary central nervous system (CNS) tumors in humans, consisting of 70% of all malignant primary brain tumors. These tumors have poor prognosis due to treatment resistance and invasiveness of residual tumor cells and recurrence after resection, characteristics that are attributed to GSCs. In this study we explored the role of miR-137 in the stemness features and self-renewal of GSCs.

We found that miR-137 was downregulated in GBM compared to normal brain specimens and in GSCs compared to NSCs. Our results of decreased expression of miR-137 in GBM is in accordance with recent reports [26].

The downregulation of miRNAs in some tumors may be mediated by CpG island hypermethylation. We found that the promoter region of miR-137 was hypermethylated in GBM as compared with normal brain specimens, which may account, at least in part, for the downregulation of miR-137 in these tumors. Similarly, the downregulation of miR-137 in squamous cell carcinoma of the head and neck, gastric cancer, bladder cancer and colorectal cancer has been also attributed to hypermethylation of pre-miR-137 promoter [27-29].

miR-137 plays an essential role in controlling the fate of embryonic NSCs. Indeed, in mouse NSCs, miR-137 regulates the dynamics between NSC proliferation and differentiation during neural development by forming a feedback regulatory loop with TLX and LSD1 and by negatively regulating cell proliferation and accelerating the neural differentiation of embryonic NSCs [25]. Another study reported increasing levels of miR-137 in mouse NSCs that were differentiated into neurons and that miR-137 induced neuronal and reduced astrocytic differentiation in mouse NSCs and glioma cells [24].

We found that the expression of miR-137 was increased in differentiated NSCs and GSCs and that miR-137 increased the neuronal differentiation of NSCs and the neuronal and some glial differentiation of the GSCs. Moreover, miR-137 decreased the self-renewal ability of the GSCs and the expression of the stem cell associated proteins Nanog, Shh, Oct4 and Sox2. Self-renewal or secondary neurosphere formation is one of the major features of cancer stem cells and has been associated with the tumorigenic capacity of these cells, rapid tumor progression and with patient clinical outcome [30]. Collectively, our results show that miR-137 can reduce the stemness signature and characteristics of GSCs by decreasing self-renewal and inducing differentiation of GSCs.

miR-137 has been reported to act as a tumor suppressor miRNA in various types of tumors. In addition, several miR-137 targets have been recently identified in different tumors including COX-2 in GBM, CDK-6 in glioma cell lines and lung cancer, CtBP1 in melanoma cells, estrogen-related receptor alpha in breast cancer, Cdc42 in gastric cancer cells, colorectal cancer cells and lung cancer and c-Met, YB1, EZH2, and MITF in melanoma cells [24, 26, 31-35]. In this study we identified RTVP-1 as a novel target gene of miR-137 by luciferase reporter assay and by demonstrating that miR-137 downregulated the mRNA and protein level of RTVP-1 in GSCs.

RTVP-1 is highly expressed in astrocytic tumors as compared to normal brains and its expression is dependent on tumor grade [36]. This protein is also involved in the regulation of the growth and survival of glioma cells by activating c-Jun-NH2-kinase and by increasing the expression of Bcl-2, rendering glioma cells more resistant to the apoptotic effect of tumor necrosis factor-related apoptosis-inducing ligand and serum deprivation [36]. RTVP-1 also plays a role in mediating the effect of SRF on glioma cell migration [37] and in the increased invasion of these cells by activating matrix metalloproteinase 2 [36].

Our finding that miR-137 targets RTVP-1 suggests that the downregulation of RTVP-1 by miR-137 mediates at least some of the tumor suppressor effects of miR-137 in GBM and specifically the inhibition of GSC self-renewal. Indeed, we found that overexpression of RTVP-1 in GSCs increased their self-renewal, whereas silencing of this gene caused a significant inhibition. Moreover, we demonstrated that overexpression of RTVP-1 plasmid lacking its 3'-UTR abolished the inhibitory effect of miR-137 on the self-renewal of GSCs. Collectively, these results indicate that the ability of miR-137 to inhibit the stemness characteristics of GSCs was at least partly mediated by targeting of RTVP-1.

We found that silencing of RTVP-1 decreased the expression of CXCR4 which has been associated with cancer stem cell characteristics in various types of tumors [38, 39]. Moreover, overexpression of CXCR4 was able to abrogate the inhibitory effect of RTVP-1 silencing on the self-renewal of the GSCs, suggesting that the decrease in CXCR4 expression mediates at least in part the inhibitory effect of RTVP-1 silencing. CXCR4 has been implicated in the control of Shh-Gli-Nanog network and the self-renewal and expression of embryonic stem cell (ESC)-associated genes in GSCs [17, 40]. A recent study identified CXCR4 as a target of the miR-302-367 cluster [17]. Our current results emphasize the important role of this protein in controlling the self-renewal and GSC characteristics downstream of multiple pathways.

The core signaling pathways involved in cancer stem cell self-renewal include the WNT, Hedgehog, Notch, BMI, PTEN, BMP and TGF-β pathways [6]. Recent studies report that in GSCs some of these pathways also involve miRNAs that can inhibit the self-renewal ability. Such pathways include the targeting of the Bmi-1 by miR-128 and the miR-302-367 cluster that drastically affects self-renewal and infiltration properties through CXCR4 repression and consequent disruption of the Hedgehog-Nanog network [17, 19]. Tumor suppressors that inhibit cancer cell proliferation, such as p53,p16INK4a and p19ARF, also inhibit stem cell self-renewal [41]. Here we identified a novel pathway that plays a role in the self-renewal ability and differentiation of GSCs. This pathway is regulated by the brain-enriched miRNA, miR-137 through direct down-regulation of its target gene RTVP-1.

Recently, it was reported that differentiation of GSCs decreases their tumorigenic potential and renders them more susceptible to different therapies [42]. Thus, the ability of miR-137 to decrease self-renewal and promote differentiation of GSCs can be employed as a novel therapeutic approach in which the oncogenic potential of the GSCs is abolished with minimal damage to normal cells in the tumor microenvironment as compared with other current therapies in GBM [43].

In summary, we found that miR-137 is downregulated in glial tumors and in GSCs and that its promoter is hypermethylated in GBM specimens. In addition, we demonstrated that the inhibitory effect of miR-137 on GSC self-renewal was at least partly mediated by decreasing the expression of the novel miR-137 target, RTVP-1 which led to the downregulation of CXCR4 expression. These results implicate miR-137 and RTVP-1 as new therapeutic targets that regulate self-renewal and differentiation of GSCs.

## MATERIALS AND METHODS

### Materials

Primary antibodies were purchased from the following companies: anti-RTVP-1 and β3-tubulin from Abnova (Taiwan) and rabbit anti-GFAP from DAKO (Denmark). PCR primers were purchased from Invitrogen (CA, USA). The CXCR4 inhibitor AMD3100 was obtained from Sigma (MO, USA). miR-137 mimic and RTVP-1 siRNA were purchased from Dharmacon (CO, USA). A lentivirus vector expressing pre-miR-137 was obtained from System Biosciences (SBI, CA, USA) and plasmids expressing RTVP-1 and luciferase-RTVP-1-3′UTR were obtained from GeneCopoeia (MD, USA).

### Human tissue specimens

Frozen human non-neoplastic brain tissue and human GBM specimens were obtained from the Department of Neurosurgery at Henry Ford Hospital. All human materials were used in accordance with the policies of the Henry Ford Health System Institutional Review Board at Henry Ford Hospital and the committee specifically approved this study. According to the IRB protocol, a written informed consent was obtained from all patients for the use of their tumor material.

### Bisulfite treatment and methylation analysis

DNA was extracted according to the DNeasy kit, spin-column standard protocol (Qiagen, Maryland USA). Methylation analysis was performed on DNA extracted from paraffin blocks of eight GBM specimens and eight normal brains. Bisulfite treatment was performed on 1μg DNA using EZ DNA methylation Gold Kit (Zymo Research, Orange, CA), according to the Sequenom® protocol (Sequenom, San Diego, CA). Final elution of C-T converted DNA was with 50μl l H_2_O.

CpG sites within mir-137 promoter sequence were searched using USCS genome browser (http://genome.ucsc.edu).

Amplicons and primers within the promoter area were designed using SequenomEpiDesigner (www.epidesigner.com). An amplicon, which covers 32 CpG sites, starting 300bp upstream and ending 167 bp downstream of the pre-miR-137 start codon was used for the methylation analysis,.

PCR amplification of amplicon was carried out with 50 ng of bisulfite-treated DNA in a total volume of 25 μl with GoTaq buffer, 200 μM dNTPs, 5 mM MgCl_2_, 10 pmol of reverse and forward primers, and 2 U of GoTaq polymerase. PCR conditions were 95°C for 10 min followed by 35 cycles of 94°C for 20 sec, 60°C for 30 sec and 72°C for 1 min, and later 3 min in 72°C. PCR product was treated with Shrimp Alkaline Phosphatase, and was subjected to in vitro transcription into RNA and T-specific cleavage by RNAse A, all according to the Mass CLEAVE protocol (Sequenom®, San Diego, CA). Samples were purified with 6mg Resin and robotically dispensed onto 384-well spectro CHIPS by Mass ARRAY Nano dispenser (Sequenom, San Diego, CA), for the Sequenom mass spectrometry detection. Results were analyzed by EpiTyper software.

### GSC cultures and differentiation

All human materials were used in accordance with the policies of the Institutional Review Board at Henry Ford Hospital. For the generation of GSCs, fresh GBM specimens were processed as previously described (44,45) and spheroids were maintained in neurosphere medium (DMEM-F12 1/1, glutamine 10 mM, HEPES buffer 10 mM and sodium bicarbonate 0.025%) supplemented with bFGF (20 ng/ml) and EGF (20 ng/ml). The GSCs were examined for the expression of CD44, Bmi-1, CD133, Musashi-1, Sox2 and nestin, self-renewal, expression of astrocytic, oligodendrocytic and neuronal markers upon plating on poly-D-ornithine in serum-containing medium and for their tumorigenic potential in nude rats.

For the differentiation studies, the NSCs and GSCs were disaggregated into small neurospheres and were plated on laminin coated plates in F12 medium supplemented with 2 % N12 (NM medium). For studying the effects of pre-miR-137, the cells were maintained in DMEM-F12 1/1 supplemented with 2% FCS and in the absence of growth factors.

### Transfection and transduction of GSCs

Transfection of GSCs with the miR-137 mimic (SMARTpool, Thermo Scientific, Lafayette, CO) was performed by siIMPORTER (Millipore, Billerica, MA) according to the manufacturer's recommendations. A lentiviral vector (System Biosciences, Mountain View, CA) expressing pre-miR-137-GFP and lentivirus vectors expressing RTVP-1 and control shRNAs or CXCR4 and RTVP-1 lacking the 3'-UTR were packaged and used to transducer the GSCs and NSCs according to the manufacturer's protocol.

### Luciferase assay

Cells were first transfected with appropriate plasmids in 12-well plates, and then were harvested and lysed for luciferase assay 48 h after transfection. Luciferase assays were performed by using a luciferase assay kit (Promega, Madison, WI) according to the manufacturer's protocol. Renilla luciferase was used for normalization.

### Neurosphere formation assay

For analyzing the ability of GSCs to form secondary neurospheres, cells were plated in 24-well plates at a density of 100 cells/well through limiting dilution and the number of neurospheres/well was determined 14 days thereafter for eight different wells. Neurospheres that contained more than 20 cells were scored as previously described (44).

### Western blot analysis:

Western blot analysis was performed as described. Equal loading was verified using an anti-β-actin or tubulin antibodies.

### Real-time quantitative PCR analysis

Total RNA was isolated using TRIzol reagent (Invitrogen, Grand Island, NY) per the manufacturer's protocol and 1 μg of RNA was used to synthesize cDNA by SuperScriptase III (Invitrogen) with random primers. To detect the mRNAs of the different genes examined, the SYBR green method was used with the following primers: Shh - Forward; TCGGTGAAAGCAGAGAAC;

reverse: AGGAAAGTGAGGAAGTCG; CXCR4 - forward: GTCATCTACACAGTCAACCTCTAC; reverse: ACCACCTTTTCAGCCAACAG; RTVP-1 - forward: GTGAAACCAACAGCCAGTGA; reverse TCTCTCCCAGTGAAGTGAAG; Oct4 - forward: ATCAGCCACATCGCCCAGCA; reverse: CCCAGCAGCCTCAAAATCCT; Sox2 forward: TGGGTTCGGTGGTCAAGTC; reverse: CGCTCTGGTAGTGCTGGGA and S12 Forward: TGCTGGAGGTGTAATGGACG, Reverse: CAAGCACACAAAGATGGGCT. Average level of S12 RNA was used as an internal control. Expression of miR-137 in cell lines or patient specimens was detected by the TaqMan stem-loop RT-PCR method. The primers and probes of the miR-137 U6 and RNU6B endogenous control for TaqMan miRNA assay were purchased from System Biosciences (Mountain View, CA).

### Statistical analysis

The results are presented as the mean values ± SE. The data of patient specimens are presented graphically with median and interquartile range noted. Data were analyzed using analysis of variance or a Student's *t* test with correction for data sets with unequal variances. Age-adjusted t-test is taken from a linear model including age as a covariate. Data were analyzed on a log 2 scale as appropriate.
